# A Fast-Growing Oleaginous Strain of *Coelastrella* Capable of Astaxanthin and Canthaxanthin Accumulation in Phototrophy and Heterotrophy

**DOI:** 10.3390/life12030334

**Published:** 2022-02-23

**Authors:** Amélie Corato, Thanh Tung Le, Denis Baurain, Philippe Jacques, Claire Remacle, Fabrice Franck

**Affiliations:** 1 Laboratory of Bioenergetics, InBios/PhytoSystems, Department of Life Sciences, University of Liege, Chemin de la Vallée 4, 4000 Liege, Belgium; amelie.corato@uliege.be (A.C.); tungrimf@gmail.com (T.T.L.); 2 Genetics and Physiology of Microalgae, InBios/PhytoSystems, Department of Life Sciences, University of Liege, Chemin de la Vallée 4, 4000 Liege, Belgium; 3 Research Institute for Marine Fisheries, 224 Le Lai Stress, Ngo Quyen District, Hai Phong City 04000, Vietnam; 4 Eukaryotic Phylogenomics, InBios/PhytoSystems, University of Liege, Chemin de la Vallée 4, 4000 Liege, Belgium; denis.baurain@uliege.be; 5 Microbial Processes and Interactions (MiPI), TERRA Teaching and Research Centre, Joint Research Unit BioEcoAgro UMRt 1158, Gembloux Agro-Bio Tech, University of Liege, Avenue de la Faculté d’Agronomie, 2B, 5030 Gembloux, Belgium; philippe.jacques@uliege.be

**Keywords:** microalga, *Coelastrella*, Chlorophyceae, carotenoids, astaxanthin, canthaxanthin, phototrophy, heterotrophy, saturated fatty acids, monounsaturated fatty acids

## Abstract

Considering the importance of microalgae as a promising feedstock for the production of both low- and high-value products, such as lipids and pigments, it is desirable to isolate strains which simultaneously accumulate these two types of products and grow in various conditions in order to widen their biotechnological applicability. A novel freshwater strain from the genus *Coelastrella* was isolated in Belgium. Compared to other *Coelastrella* species, the isolate presented rapid growth in phototrophy, dividing 3.5 times per day at a light intensity of 400 µmol·m^−2^·s^−1^ and 5% CO_2_. In addition, nitrogen depletion was associated with the accumulation of astaxanthin, canthaxanthin, and fatty acids, which reached ~30% of dry weight, and a majority of SFAs and MUFAs, which are good precursors for biodiesel. This strain also accumulated astaxanthin and canthaxanthin in heterotrophy. Although the content was very low in this latter condition, it is an interesting feature considering the biotechnological potential of the microalgal heterotrophic growth. Thus, due to its rapid growth in the light, its carotenogenesis, and its fatty acids characteristics, the newly identified *Coelastrella* strain could be considered as a potential candidate for biorefinery purposes of both low- and high-values products.

## 1. Introduction

Astaxanthin, a ketocarotenoid, is one of the pigments with the highest biotechnological potential [1]. Because of its molecular structure [2], it shows powerful antioxidant and coloring properties [3]. In addition, astaxanthin has anti-inflammatory properties, probably caused by modulating redox signaling events [4], and has been considered to have beneficial effects on various diseases, such as cardiovascular ones [2]. Compared to carotenes and other xanthophylls, it presents a better bioavailability due to its esterification [5]. It is present in cosmetics, food, and feed products [2,5]. The main source of astaxanthin is synthetic, produced from petroleum by chemical industries [2]. However, natural astaxanthin may be preferred because it contains more biologically assimilable forms than the synthetic version [1]. The sale value of astaxanthin varies between USD 2,000 and USD 15,000·kg^−1^, with natural astaxanthin being sold at higher prices [2]. The world market is constantly growing and was estimated to be USD 600 million in 2021 [2].

Astaxanthin is produced by a limited number of organisms, of which the microalga *Haematococcus pluvialis* is the main source. This green microalga belongs to the Chlorophyceae class, and is the best domesticated producer of astaxanthin, which is commercially exploited [3].

*H. pluvialis* mainly accumulates astaxanthin, starting with the oxidation of β-carotene [6,7]. Astaxanthin accumulation typically comprises a two-stage process: in the first stage (growth stage), biomass accumulates, and in the second phase (red stage), carotenogenesis is triggered by the presence of reactive oxygen species (ROS), induced by applying various stresses such as those combining nutrient starvation (nitrate/phosphate) and high light or salt stress [8]. In addition, astaxanthin accumulation can also be induced by the addition of agents that trigger ROS generation, such as Fe^++^, responsible for hydroxyl radical generation from an iron-involved Fenton reaction [9].

Screening for new carotenogenic algae suitable for commercial development is needed in order to increase the panel of possibilities to obtain better astaxanthin productivities or extraction yields. In that respect, two other Chlorophycean green algae, *Chromochloris zofingiensis* (former name *Chlorella zofingiensis*, [10]) and *Coelastrella* sp., are emerging as promising species [11,12,13]. Both of them produce canthaxanthin in addition to astaxanthin, an intermediate of the biosynthesis pathway of astaxanthin, with similar commercial value to this pigment, due to its antioxidant, anti-inflammatory and colorful properties [5].

*C. zofingiensis* is interesting because of its ability to produce astaxanthin and canthaxanthin in heterotrophic conditions [14]. Compared to phototrophic cultivation, heterotrophic growth of microalgae can usually increase cell concentration, biomass densities, and volumetric productivities. Indeed, heterotrophic growth is not limited by the inefficient light penetrance and distribution inside photobioreactors or open ponds, which result in low cell concentrations. The cost of the downstream processing associated with the volume of water to be treated is therefore reduced. In addition, the buying and running costs of photobioreactors are high, while the classical fermenters used for growth in the dark are cheaper to buy and maintain. However, heterotrophic growth presents several drawbacks, such as the costs linked to the organic substrates and the fact that some light-induced metabolites cannot be produced in the dark (reviewed in [15]). To solve these problems, growth on agricultural byproducts enables the reduction of costs, and ROS-inducers, such as H_2_O_2_, can be used for astaxanthin accumulation in the dark [15].

The genus *Coelastrella* belongs to the Chlorophyta phylum, to the Sphaeropleales order, and to the Scenedesmaceae family, and it is peculiar because the cell wall, composed of two layers—the internal wall being made of cellulose, and the external wall being made of sporopollenin, presents meridional ribs connecting the two poles of the cell [16,17,18]. Some strains/species with biotechnological relevant properties, such as the presence of astaxanthin and canthaxanthin, have been described, mainly selected in Asia or East Europe (e.g., [8,13,19,20]. Others also accumulate other photoprotective compounds, such as mycosporine-like amino acids which are effective against UV-A [21]. Amongst them, a few simultaneously accumulate both carotenoids and fatty acids [20]. Since multiple products can be exploited from a single cycle of growth (reviewed in [22]), this increases their commercial value. Fatty acids can be interesting for two reasons, depending on their composition. Saturated (SFAs) and monounsaturated fatty acids (MUFAs) are good precursors for biodiesel, while long-chain polyunsaturated fatty acids (PUFAs) are interesting for human health because they contribute to the reduction of the risk of cardiovascular diseases (reviewed in [23]). *Coelastrella* accumulate one or the other type of fatty acids, depending on the strain considered [12,20].

A microalgal strain was isolated in the Walloon region of Belgium. The strain divided rapidly in phototrophy and turned red under stress conditions. These two characteristics prompted us to identify it molecularly. After its identification as a *Coelastrella* isolate, the objectives of the work were (1) to characterize the growth in different cultivation conditions (the growth phase), and (2) to determine the impact of the growth phase condition on pigment accumulation and fatty acid composition profile and content in different stress conditions, both in the light and in the dark. The results presented here showed that the *Coelastrella* isolate accumulates both astaxanthin and canthaxanthin, as well as fatty acids mainly enriched with SFAs and MUFAs, during the reddening in the light. This isolate also accumulates astaxanthin and canthaxanthin in heterotrophy. It can thus be concluded that this isolate is biotechnologically interesting because it grows fast and accumulates two types of valuable compounds exploitable in one growth cycle. In addition, its capacity to grow in heterotrophy widens its applicability with the condition that the pigment content could be increased.

## 2. Materials and Methods

### 2.1. Microalga Strain, Medium and Culture Conditions

The microalga strain *Coelastrella* sp. S6 was sampled from an open pond in the Liège region (Belgium). Axenicity was reached after several subculturings on mBBM (“Bold Modified Basal Freshwater Nutrient Solution”, Sigma-Aldrich, Saint-Louis, MI, USA) agar plates supplemented with ampicillin at 5 µg·mL^−1^ [24]. The axenic cells were then maintained at 25 °C on mBBM-3N added to NaNO_3_ 750 mg·L^−1^ agar plates, to avoid any nitrate depletion [25,26], under a weak light-intensity of 5 µmol·m^−2^·s^−1^. The 3N medium contains three times more nitrate compared to the BBM medium and is routinely used [27].

Liquid cultures were maintained axenically in phototrophic conditions, under continuous fluorescent light at 50 µmol·m^−2^·s^−1^, at 25 °C, in a home-made, so-called Bold-Pipes “BP” medium consisting of (per liter): 750 mg NaNO_3_; 75 mg K_2_HPO_4_; 175 mg KH_2_PO_4_; 75 mg MgSO_4_·7H_2_O; 25 mg CaCl_2_·2H_2_O; 25 mg NaCl; 4.97 mg Na_2_EDTA; 0.58 mg FeCl_3_·6H_2_O; 0.012 mg CoCl_2_·6H_2_O; 0.25 mg MnCl_2_·4H_2_O; 0.024 mg Na_2_MoO_4_·2H_2_O; and 0.03 mg ZnCl_2_. The pH was maintained at 7 with 6.04 g·L^−1^ of piperazine-N,N′-bis(2-ethanesulfonic acid) (PIPES) buffer. The use of this buffer also avoids cell-aggregate formation in liquid culture. The flasks were mildly agitated on an orbital shaker (SK-71 Lab Companion) under air.

### 2.2. Optical Microscopy and SEM Analysis

The microalga morphology was analyzed using a microscope (Nikon Eclipse NiU) with total magnification of 100×, 200×, and 600×. Photomicrographs were taken with a Nikon Digital Sight DS-Ri1 camera attached to the microscope. A Neubauer chamber was used to calibrate the photomicrographs. The cells were measured by analyzing the photomicrographs with the ImageJ program (ImageJ 1.49). A total of 910 cells were measured for analyzing the cell diameter of the green cells, and 1615 cells for analyzing the cell diameter of the red cells. For scanning electron microscopy (SEM) analysis, green cells were diluted in water. A drop was deposited on the SEM support and dried overnight in an oven (60 °C). To analyze the red cells, the pellet was freeze-dried overnight. The prepared samples were covered by 20 nm of gold. The cells were then examined with an ESEM electronic microscope (Philips ESEM XL30 FEG) with an acceleration voltage of 5 kV.

### 2.3. Molecular Identification and Phylogenetic Analyses

The DNA extraction was performed using a modified version of Newman [28]. Microalgal cells (1 × 10^8^ cells) were centrifuged at 13,000× *g* for 2 min and the supernatant was discarded. The pellets were frozen in liquid nitrogen, and the cells were disrupted using a cryo-mixer and a metallic ball (Retsch mixer MM200); the conditions of mixing were 25 Hz for 5 min. The DNA sequence encoding Internal Transcribed Spacer 2 *(ITS2)* gene was amplified by polymerase chain reaction (PCR), using primers 5′-GGAAGTAAAAGTCGTAACAAGG-3′ and 5′-TCCTCCGCTTATTGATATGC-3′ [29]. The PCR products were sequenced on both strands (Genewiz, Leipzig, Germany).

The *ITS2* gene sequence was assembled using the CAP3 program [30] (and deposited under the accession MW915611.1). It was then compared with BLAST (blastn) [31] to the GenBank database [32]. A total of 260 sequences with an identity higher than 90% to the query were extracted and a multiple alignment was realized using MAFFT v7.310 [33,34]. The alignment was then refined manually using the SeaView editor (v4, [35]). The phylogenetic tree, based on the maximum likelihood criterion, was inferred with PhyML [36], under a GTR+Γ4 model [37] and a heuristic search using NNI rearrangements. The starting tree was created using the BIONJ algorithm, and the statistical support resulted from aLRT tests [38]. The phylogenetic tree obtained was automatically annotated using format-tree.pl (from Bio-MUST-Core package; D. Baurain, unpublished; https://metacpan.org/dist/Bio-MUST-Core, accessed on 18 February 2022) and the NCBI Taxonomy. Finally, it was visually arranged using iTOL [39].

### 2.4. Growth and Carotenogenesis Conditions

A table of the conditions used for the growth (growth phase, phase 1) and the carotenogenesis (red phase, phase 2) is provided in Table 1. Phase 1 of phototrophic growth was performed for 7 days, in “BP” medium, under continuous fluorescent light at an intensity of 400 µmol·m^−2^·s^−1^ with (“CO_2_”) or without (“Air”) supplementation of the incoming air with 5% CO_2_. Phase 1 of heterotrophic growth was performed in complete darkness, using “BP” medium added to 10 g·L^−1^ glucose for 10 days. Cultures were seeded at a dry weight of 0.25 g·L^−1^ in 500 mL flasks containing 100 mL of medium and mildly agitated (3–5 g) on an orbital shaker (SK-71 Lab Companion). The gases (air or CO_2_) were injected using a sterile filter on the top of the flasks at a flow rate of 50 mL·min^−1^·flask^−1^.

Phase 2 of carotenogenesis was stress-induced under phototrophic or heterotrophic conditions in cultures adjusted to an initial dry biomass of 0.88 g·L^−1^. The phototrophic stress-inducing condition was realized under 5% CO_2_ and made of the combination of exposure to high light intensity (400 µmol·m^−2^·s^−1^) and nitrogen starvation (transferring the algae in “BP” medium without NO_3_^−^) for both “Air” and “CO_2_” conditions. Heterotrophic stress-inducing conditions consisted in nitrogen starvation only (Condition 1) or on nitrogen starvation combined with ROS induction (Condition 2), both in the presence of 10 g·L^−1^ glucose. For condition 2, ROS induction was triggered by the addition of 500 µM menadione sodium bisulfite (MSB) (every 4 days), 1 g·L^−1^ sodium acetate (every 2 days), and 18 µM ferrous ion (Fe^2+^) (daily). All the experiments were carried out in triplicate, and all the data are provided as mean ± standard deviation of the three, independent biological replicates.

### 2.5. Algal Growth Measurements

The algal growth was monitored daily by optical density (OD) measurements and then reported in terms of dry biomass, using an “OD/Biomass” correlation [40]. The optical density was measured at a wavelength of 750 nm with a spectrophotometer UV-visible Ultrospec1000 (Pharmacia Biotech) in cuvettes of an optical path of 1 cm. The dry biomass was estimated by centrifuging 10 to 15 mL of culture (3 min at 3000× *g*). The samples were washed with distilled water, and the pellets were deposited on filter papers (Glass Microfiber Filters, GF/C, Whatman). The biomass was dried for 48 h in an oven (70 °C), and the filter was weighed. Dry biomass was determined by subtracting the two weights.

The maximum specific growth rate (µmax), expressed in h^−1^, was calculated during the exponential growth phase on the basis of a linear fit of ln(OD)_t_ as a function of time, where OD_t_ was measured at 750 nm. The maximal volumetric biomass productivity (Vmax), expressed in g·L^−1^·day^−1^, was calculated from the part of the OD growth curve where the growth rate was maximal. The statistical tests were realized using Student’s *t*-test.

### 2.6. Nitrate and Glucose Quantification

Nitrate concentration in the medium was quantified using a colorimetric method developed by [41]. Culture supernatants were harvested by centrifugation at 16,000× *g* for 3 min and diluted to obtain an estimated nitrate concentration range between 0 and 2.5 mM. In parallel, the standard solutions of 0, 0.5, 1, 1.5, 2, and 2.5 mM NaNO_3_ were prepared. A quantity of 20 µL of salicylic acid 2.5% (in H_2_SO_4_ 98%) was added to 20 µL of sample and incubated for 20 min at room temperature. A quantity of 500 µL of NaOH 3.8M was added and 100 µL of the samples was analyzed at 405 nm using a spectrophotometer Lamba 265 (PerkinElmer) in 96-well plates.

Glucose and acetate concentrations in the medium were quantified by high-performance liquid chromatography (HPLC, Shimadzu), using a refractor index detector [42]. Supernatants were loaded on an ion exclusion column SUPELCOGEL C610-H (6% Crosslinked, 9 μm particle size, L × I.D. 30 cm × 7.8 mm, Sigma-Aldrich). The eluent was H_3_PO_4_ 0.1%, the mode was isocratic with a flow of 0.5 mL·min^−1^. The elution was performed for 35 min at a temperature of 35 °C. The quantification was based on the peak area, compared with a standard solution of glucose and acetate 2 g·L^−1^.

### 2.7. Chlorophylls, Primary Carotenoids (Neoxanthin, Violaxanthin, Antheraxanthin, Lutein, Zeaxanthin and β-Carotene), Astaxanthin and Canthaxanthin Determination

Cells were centrifuged at 16,000× *g* and 500 µL of dichloromethane/methanol (1:3, *v*/*v*) was added to the pellets. Cells were disrupted using glass beads and agitation in a TissueLyser II (Qiagen, Venlo, Netherlands), for 5 min at 25 Hz. The extraction was conducted over 30 min using a Vibrax VXR (Ika). The samples were centrifuged at 13,000× g for 2 min, and a second extraction was carried out over 15 min, with 250 µL of extraction solvent. Pigments were analyzed using reverse-phase HPLC with a photodiode array (PDA) detector (Shimadzu, Kyoto, Japan). The separation was conducted by reverse-phase on a CORTECS C18 column (90 Å pore size, 2.7 µm particle size, 4.6 mm × 150 mm, 3/pkg). The analyses were realized at 25 °C with a flow rate of 1 mL·min^−1^. The eluents used were: 75% acetonitrile + ammonium acetate 50mM (A), 90% acetonitrile (B), and 100% ethyl acetate (C). The gradient was: 0 min—100% A; 2.5 min—100% B; 3.1 min—90% B + 10% C; 8.1 min—65% B + 35% C; 13.5 min—40% B + 60% C; 17 min—100% C; 19 min—100% A; 25 min—100% A. The data were analyzed with LabSolution software (Shimadzu), and the quantification based on the peak area at 430 nm, in comparison with standard solutions of chlorophylls (*a* and *b*), astaxanthin, canthaxanthin, and primary carotenoids (neoxanthin, violaxanthin, antheraxanthin, lutein, zeaxanthin, and β-carotene) (DHI Lab Products, Horsholm, Denmark). These standards allow external calibration to be made using calibration curves [43].

### 2.8. Lipids and FAMEs Determination

The total lipid fraction was quantified by gravimetry, and lipid extraction was conducted based on a method described by Bligh and Dyer [44], as follows, on 60 mg dry weight (DW) of microalgae. Microalgae were harvested and lipid extraction was realized by four successive steps of cell disruption (2 × 5 min and 2 × 3 min, at 25 Hz) in extraction solvent CHCl_3_/MeOH (2:1) (2 × 1.5 mL et 2 × 1 mL) in the TissueLyser II (Qiagen). The four cell disruption steps (2 × 5 min and 2 × 3 min) were followed by 30 min agitation on Vibrax and supernatant recovery. All the supernatants were collected, 0.9% NaCl was added (three times) to remove proteins and polar compounds from the organic phase. This last one was then recovered in aluminum caps of 5 mL. The lipids were dried overnight at room temperature, followed by 2 h in the oven (70 °C), and the caps were weighed. Total lipid extracts were determined by subtracting the two weights.

The fatty acid methyl esters (FAMEs) fraction was determined on 3 mg DW of microalgae. A quantity of 500 µL of CHCl_3_/MeOH (2:1) was added to the pellets, and cells were disrupted using a TissueLyser II (Qiagen), for 5 min at 25 Hz. Extraction was pursued for 15 min on a Vibrax VXR (Ika). This first extraction step was repeated twice by addition of 250 µL extraction solvent, 15 min agitation on Vibrax, and supernatant harvesting. The supernatants were pooled together, and 800 µL was recovered, washed with 1 mL of 0.9% NaCl, and the chloroform phase was harvested and dried overnight at room temperature. The dried lipids were resuspended in 1 mL methylation reactive [33% methanolic HCl 3N, 67% MeOH and 10 µg·mL^−1^ butylated hydroxytoluene (BHT)] in sealed vials, and transesterification was then performed at 70 °C, for 1 h. The FAMEs were then transferred in 800 µL of 100% heptane via four consecutive liquid–liquid extractions. They were quantified using gas chromatography (GC), and a flame ionization detector (FID, Shimadzu), as mentioned in [45]. The FAME separation was performed using SGE BPX70 column (30 m × 0.25 mm × 0.25 μm) with helium as the carrier gas. The temperature gradient was 120 °C to 240 °C at a speed of 4 °C·min^−1^. A quantity of 1 µL of sample was used and injection was made in ‘split’ mode with a ratio of 10. The quantification of FAMEs was based on the peak area, using an external calibration curve realized based on a FAMEs mix, suitable for microalgae fatty acids determination (Supelco37, Sigma-Aldrich) (no internal standards).

## 3. Results

### 3.1. Strain Identification

**Figure 1:**
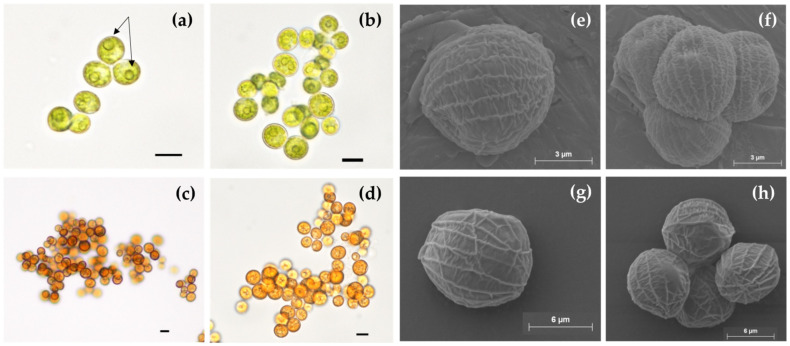
Micrographs of green and red *Coelastrella* sp. S6 cells obtained by optical and scanning electron microscopy. Arrows indicate pyrenoids; (**a**,**b**,**e**,**f**): “green” culture at the end of exponential phase; (**c**,**d**,**g**,**h**): “red” culture after 4 days of stress (a combination of high light exposure and nitrogen starvation). Scale for (**a**–**d**): 10 µm; scale for (**e**) and (**f**): 3 µm; and for (**g**) and (**h**): 6 µm.

The microalgal isolate was sampled from an open pond in the Liège region (Belgium) and reached axenicity after several subculturings on mBBM agar plates supplemented with ampicillin. A light microscope and a lab binocular were used to observe the algal cells and colonies and ensure axenicity (Figure 1a,b). The isolate showed the ability to turn red in response to high light and nutrient-starvation stress conditions (Figure 1c,d). “Green” cells had 8.7 ± 1.5 µm diameter. They were spherically shaped, non-flagellated, and possessed a voluminous pyrenoid (black arrows, Figure 1a). They divided by forming 4 to 16 autospores (Figure 1f). The “red” cells had 9.3 ± 1.6 µm diameter (Figure 1c,d). The cell surface ornamentations of the isolate were visible using electron scanning microscopy (SEM) (Figure 1e–h). The cells possessed meridional ribs that connected one pole of the cell to the other and formed polar thickenings at each of them. These ornamentations, i.e., meridional ribs, and morphological features are characteristics of the *Coelastrella* genus [16,46,47].

**Figure 2:**
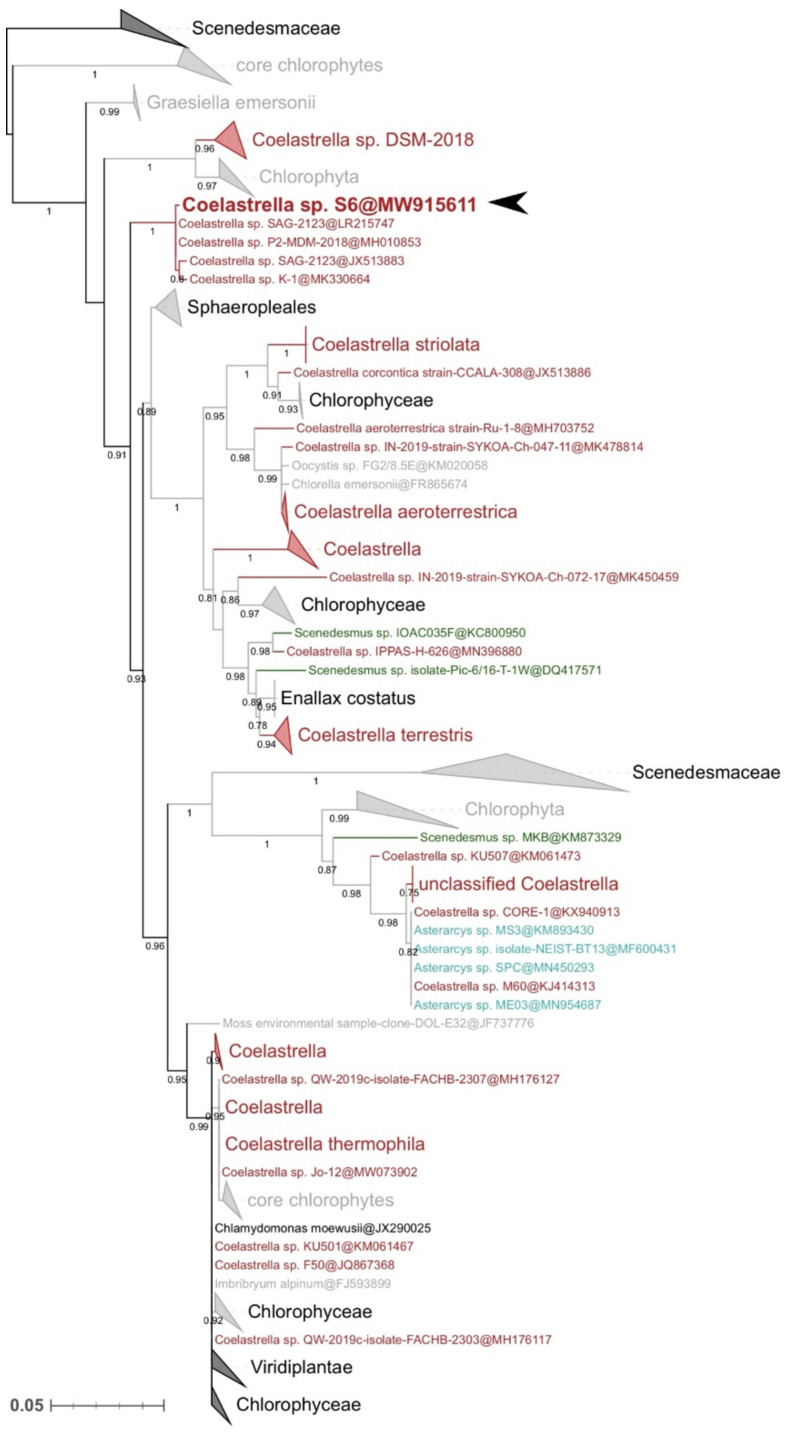
Maximum likelihood phylogenetic tree built from GenBank *ITS2* sequences with more than 90% nucleotide identity to the studied isolate. Numbers on branches represent aLRT support values (only those greater than 0.75 are shown for clarity). Strains of the genus *Coelastrella* are indicated in red, whereas other Scenedesmaceae genera are in other colors. The position of *Coelastrella* sp. S6 is highlighted by a black arrowhead. Scale is in substitutions per site.

Using PCR amplification and DNA sequencing, the *ITS2* sequence (693 bp) was determined (accession no.: MW915611.1). The maximum likelihood tree, built on the dataset containing public sequences having at least 90% identity with the target sequence, is shown in Figure 2. The isolate is grouped in a cluster with other algae from the genus *Coelastrella*, with high statistical support (bootstrap = 1), and displays a strong relationship with *Coelastrella* sp. SAG-2123 (LR215747 and JX513883), *Coelastrella* sp. P2-MDM-2018 (H010853) and *Coelastrella* sp. K-1 (MK330664) (aLRT = 1.0). Although the *Coelastrella* genus does not appear monophyletic [16,17,48], the morphological and phylogenetic analyses suggest that the isolate is strongly related to the genus *Coelastrella*, and it was thus named *Coelastrella* sp. S6 (Figure 2).

### 3.2. Characterization of the Coelastrella sp. S6 Isolate in Terms of Growth, Pigments and Fatty Acids Profiles and Accumulation

#### 3.2.1. Growth Analysis during the Growth Phase (Phase 1) in Phototrophic and Heterotrophic Conditions

The carotenogenic characteristics of the *Coelastrella* sp. S6 isolate were evaluated using two-stage experimental protocols, both in the light and in the dark. In the first growth stage (also called phase 1), cells were grown on complete media (no nutrient limitation at the start of the culture) to favor growth either in the light (phototrophy) or in the dark (heterotrophy). In the second “red” phase (also called phase 2), a stress was applied: nitrogen deprivation combined with high light exposure or, in the dark, nitrogen deprivation combined (or not) with addition of menadione sodium bisulfite (an ROS producer) and acetate.

**Figure 3:**
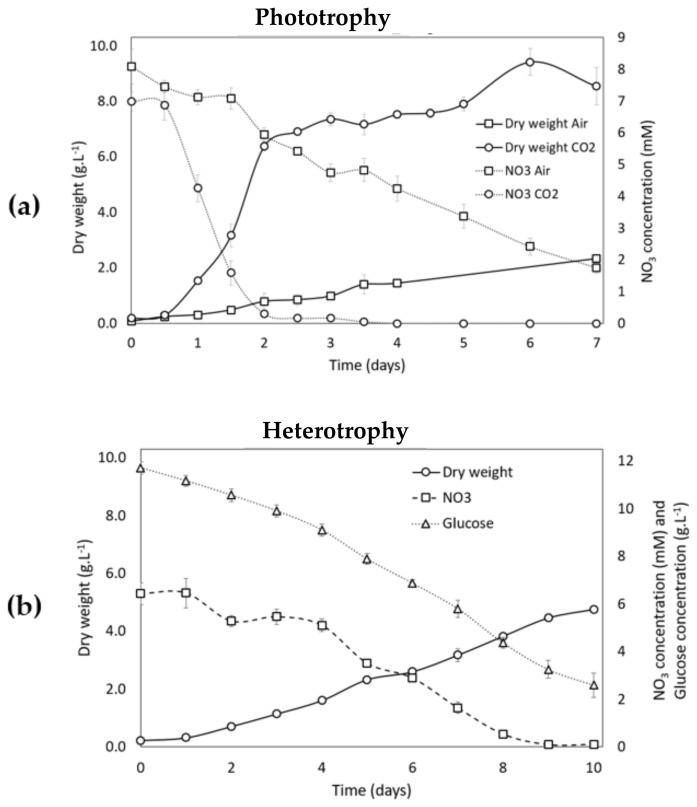
Evolution of dry weight of *Coelastrella* sp. S6 cells and nutrient concentration in the medium during the growth phase (phase 1) in different conditions. (**a**) Dry weight of cells in phototrophic conditions for cultures under “Air” condition (squares and continuous line) or “CO_2_” condition (circles and continuous line). Nitrate concentration in the medium from “Air” condition (squares and dotted line) or “CO_2_” condition (circles and dotted line). (**b**) Dry weight of cells in heterotrophic conditions (circles). Nitrate (squares) and glucose (triangles) concentration in the medium. The data are reported as the mean ± standard deviation from three independent biological replicates.

For the phototrophic growth phase, cells were cultivated for seven days on complete medium at 400 μmol photons·m^−2^·s^−1^ under injected air (“Air” condition) or 5% CO_2_ (“CO_2_” condition) as described in the Materials and Methods Section and Table 1. It has already been shown for other green microalgae that a high light intensity of 400 μmol photons·m^−2^·s^−1^ allows for the obtaining of a higher biomass productivity and specific growth rate than do lower light intensities [49,50]. As shown in Figure 3a, biomass accumulated after seven days of growth in the “CO_2_” condition was four times higher (8.53 ± 0.68 g DW·L^−1^) than in the “Air” condition (2.33 ± 0.06 g DW·L^−1^) at the end of the green phase. The maximum growth rate (µmax) was 0.102 ± 0.002 h^−1^ (doubling time of 6.8 ± 0.30 h) in the presence of CO_2_, which was about twice as high as in the “Air” condition (µmax = 0.045 ± 0.002 h^−1^, doubling time of 15.4 ± 0.75 h). CO_2_ has already been shown to increase growth performances of the carotenogenic microalga *H. pluvialis* [51,52] and other green microalgae, such as *Chlorella vulgaris* and *Scenedesmus obliquus* [53,54,55]. During the growth phase, the increase of biomass was accompanied by a decrease in nitrate concentration in the culture medium (Figure 3a). Under the “CO_2_” condition, nitrate was likely the limiting factor since the stationary phase was nearly reached upon nitrate exhaustion around day 2, before the cells were collected for the carotenogenesis (“red”) phase at day 7. In contrast, nitrate was still present when the cells were collected for the second phase under the “Air” condition.

Heterotrophic growth was performed in the presence of glucose (10 g·L^−1^) as organic carbon source under injected air as described in the Materials and Methods Section (Figure 3b). The growth was followed for 10 days, when biomass reached 4.8 g·L^−1^. The µmax was 0.018 ± 0.000 h^−1^ (doubling time of 38.5 h, 0.81 division per day), which was much lower than in the light conditions (see above). The growth slowed down after 9 days when nitrate was close to exhaustion (Figure 3b). Glucose was not exhausted after the 10 day growth phase, and the yield of glucose conversion to biomass (Yx/s) was 0.48 ± 0.01 g DW·g^−1^ Glucose (Figure 3b). This value is consistent with the theoretical conversion value (0.5 gDW·g^−1^ Glucose) and with values obtained for other green microalgae [14,56].

#### 3.2.2. Growth Analysis during Carotenogenesis in Phototrophic Conditions

**Figure 4:**
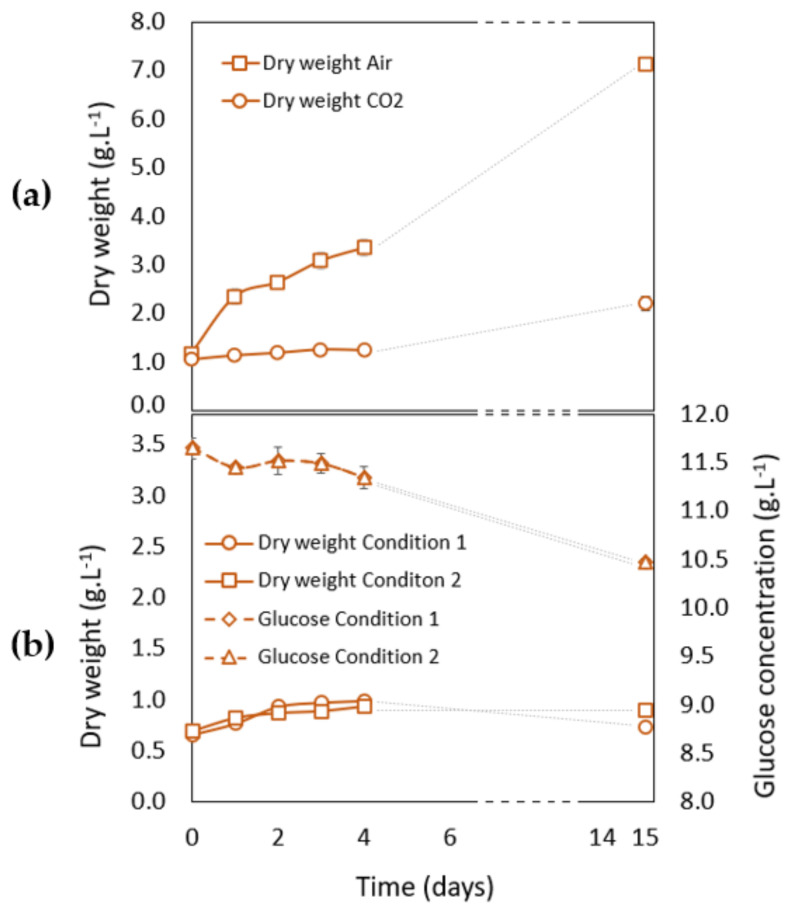
Evolution of dry weight of *Coelastrella* sp. S6 cells during “red” phase (phase 2) in different conditions: (**a**) Dry weight of cells in phototrophic stress conditions for cultures after a growth phase under “Air” (squares) or “CO_2_” condition (circles); (**b**) Dry weight of cells in heterotrophic stress conditions. Condition 1: Circles, Condition 2: Squares. Evolution of glucose concentration in the medium. Condition 1: Diamonds, Condition 2: Triangles. The time scale has been interrupted between days 6 and 14. The data are reported as the mean ± standard deviation from three independent biological replicates.

After 7 days of culture in either of the two different growth conditions (“Air” or “CO_2_”), the carotenogenesis (phase 2) was induced in nitrate-deficient medium, under high light intensity (400 µmol·m^−2^·s^−1^) and in presence of 5% CO_2_ (Table 1). During the 15 days of carotenogenesis induction, the biomass content fluctuated differently, depending on the growth phase condition (“Air” or “CO_2_”) (Figure 4a). On the one hand, almost no increase of dry weight was observed after the growth phase in the “CO_2_” condition. This is a classical feature and a consequence of nitrate starvation. On the other hand, surprisingly, the dry weight increased after the green “Air” condition and became 4 times higher than after the growth phase in the “CO_2_” condition. Thus, despite the absence of nitrogen in the medium in phase 2, the cells coming from the growth “Air” condition assimilated inorganic CO_2_ contrary to the cells cultivated under CO_2_ during the growth phase. As nitrogen is also needed for the growth, this suggests that cells were able to accumulate some nitrogen storage compounds during growth in the “Air” condition, which could then be subsequently used during the stress phase in combination with inorganic CO_2_ to increase biomass. As already mentioned, this is not the case for cultures grown in the presence of CO_2_ during the growth phase (phase 1) which are in a resting stage concomitant with the activation of carotenogenesis [6]. We hypothesize that this resting stage of the “CO_2_” culture was probably even present before the induction of carotenogenesis at day 7, since nitrate was already naturally depleted at day 2 of the culture under CO_2_ (Figure 3a). An increase of dry weight after nitrate depletion has already been observed in *Scenedesmus* and in *Coelastrella* species [13,57] but has not been discussed in detail.

#### 3.2.3. Growth Analysis during Carotenogenesis in Heterotrophic Conditions

As heterotrophic growth gains interest for biotechnological applications (see introduction), carotenogenesis of *Coelastrella* sp. S6 has also been induced in full dark conditions (Figure 4b). Two conditions have been tested. In Condition 1, nitrate starvation was realized in the presence of glucose, and in Condition 2, in the presence of both glucose and acetate, the addition of an ROS inducer, the menadione sodium bisulfite, and of Fe^2+^ to activate the Fenton reaction (Table 1). This last condition has already been employed to trigger carotenogenesis of *H. pluvialis* [9]. One g·L^−1^ of acetate was added every two days during the carotenogenesis. Acetate concentration in the medium decreased from 1.0 ± 0.0 g·L^−1^ at day 0 to 0.5 ± 0.0 g·L^−1^ at day 2; the second addition at day 3 led to a concentration in the medium of 1.4 ± 0.0 g·L^−1^, which decreased to 1.2 ± 0.0 g·L^−1^ at day 4. Then, we assume that the acetate consumption progressively decreased since, at day 15, its concentration reached 8.3 ± 0.1 g·L^−1^. Results of Figure 4b showed that the two conditions led to growth arrest and that almost no glucose had been consumed when cells were collected for analysis after 15 days of stress in either Condition 1 or Condition 2.

#### 3.2.4. Chlorophyll and Primary Carotenoid Contents of *Coelastrella* sp. S6 in Phototrophic Conditions

**Figure 5:**
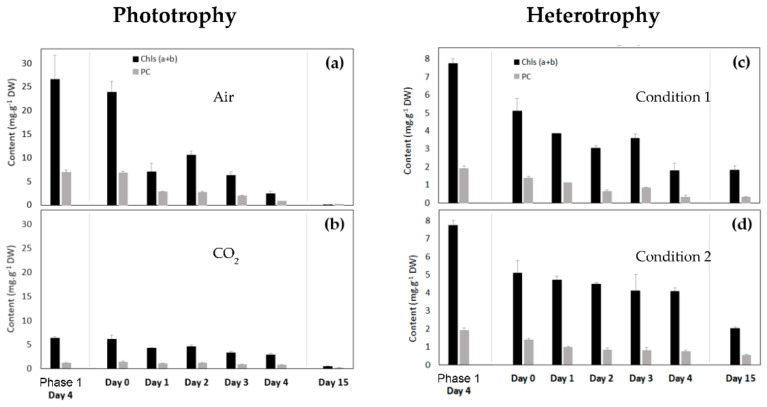
Evolution of chlorophyll and primary carotenoid content of *Coelastrella* sp. S6 cells at day 4 of the growth phase (phase 1) and at days 0, 1, 2, 3, 4, and 15 of the stress phase (phase 2), in different conditions. Chls = Chlorophylls, PC = Primary Carotenoids (see text for details). Phototrophic conditions: (**a**) Cells were cultivated under “Air” during the growth phase; (**b**) Cells were cultivated under “CO_2_” during the growth phase. Heterotrophic conditions: (**c**) Condition 1; (**d**) Condition 2. The data are reported as the mean ± standard deviation from three independent biological replicates.

The evolution of the primary pigment content at day 4 of the growth phase (phase 1) and during the “reddening” phase (i.e., carotenogenesis, phase 2), normalized on dry weight (DW), is presented in Figure 5a and b using the methodology described in the Section 2.7 of the Materials and Methods Section. Two classes of primary pigments were considered: Chlorophylls (Chls = chlorophylls a + b) and Primary Carotenoids (PC = neoxanthin + violaxanthin + antheraxanthin + lutein + zeaxanthin + β-carotene). Primary pigment content did not change between day 4 of the growth phase and day 0 of the reddening phase, whatever the cultivation condition of the growth phase (Air or CO_2_) (Figure 5a,b). However, the presence of CO_2_ had an impact on the pigment content. Indeed, the growth phase under “Air” led to a higher primary pigment content (Chls = 23.91 ± 2.24 mg·g^−1^ DW and PC = 6.87 ± 0.52 mg·g^−1^ DW) than with CO_2_ supplementation (Chls = 6.15 ± 0.84 mg·g^−1^ DW and PC = 1.36 ± 0.27 mg·g^−1^ DW).

This difference could be related in part to the establishment of nitrate starvation of the culture under CO_2_ already during the growth phase (Figure 3a) since nitrate starvation leads to a decrease in primary pigment content [6]. No matter the growth condition, the content in primary pigments decreased during the carotenogenesis process to reach low values after 15 days of reddening (“Air” condition: Chls = 0.022 ± 0.001 mg·g^−1^ DW and PC = 0.18 ± 0.05 mg·g^−1^ DW; “CO_2_” condition: Chls = 0.40 ± 0.19 mg·g^−1^ DW and PC = 0.14 ± 0.06 mg·g^−1^ DW) due to the nitrate starvation in both conditions [58].

#### 3.2.5. Chlorophyll and Primary Carotenoid Contents of *Coelastrella* sp. S6 in Heterotrophic Conditions

The evolution of the primary pigment content at day 4 of the growth phase in heterotrophy and during the reddening phase is presented in Figure 5c,d. These contents were higher at day 4 of the growth phase than at day 0 of the reddening phase, which could be related to a nitrate deficiency already established during the growth condition (at day 9 of the culture, Figure 3b). As with the phototrophic conditions, these contents decreased during the reddening phase.

#### 3.2.6. Astaxanthin and Canthaxanthin Contents of *Coelastrella* sp. S6 during Carotenogenesis in the Light and in the Dark

**Figure 6:**
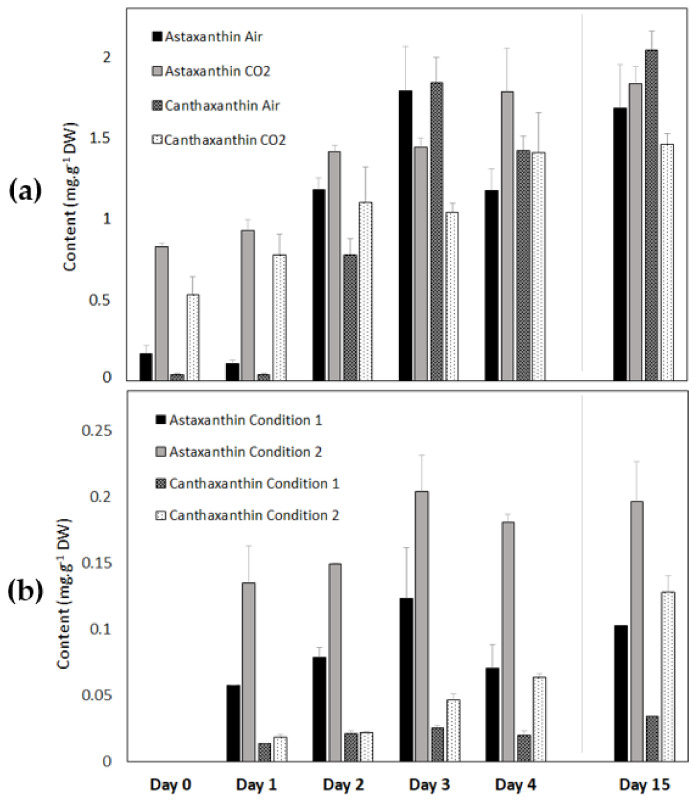
Astaxanthin and canthaxanthin content evolution during stress in different conditions: (**a**) Phototrophic conditions (after a growth phase under “Air” and under “CO_2_”); (**b**) Heterotrophic conditions (Condition 1 and Condition 2).

The astaxanthin and canthaxanthin contents were determined using the methodology described in Section 2.7 of the Materials and Methods Section. At the beginning of carotenogenesis in the light (day 0–day 1 of the reddening phase), the astaxanthin and canthaxanthin contents normalized on dry weight were higher for the “CO_2_” condition, which is consistent with a pre-activation of carotenogenesis (Figure 6 and Table 2). In contrast, the astaxanthin and canthaxanthin content increased during the reddening, subsequent to a growth phase in the “Air” condition. After 15 days, it reached about the same values as after the “CO_2_” condition. Thus, the final astaxanthin and canthaxanthin contents at the end of the red phase were not dependent on CO_2_ availability during the green phase. However, the volumetric contents (i.e., yields) of secondary carotenoids (Table 2), expressed in mg·L^−1^, were higher in the “Air” condition (respectively, three and five times higher for astaxanthin and canthaxanthin) than in the “CO_2_” condition. This was due to the previously discussed increase of dry weight occurring during the carotenogenesis for the “Air” condition (Figure 4a).

Astaxanthin and canthaxanthin accumulation in the dark was analyzed in Figure 6b and Table 2. Condition 2 had a positive impact on the biosynthesis of astaxanthin and canthaxanthin and allowed the doubling of their contents compared to Condition 1 although the values were around 10 times lower than in the light (~1.8 vs. ~0.2 mg·g^−1^ DW for astaxanthin and ~1.5 vs. ~0.1 mg·g^−1^ DW for canthaxanthin in the light and in the dark, respectively).

**Figure 7:**
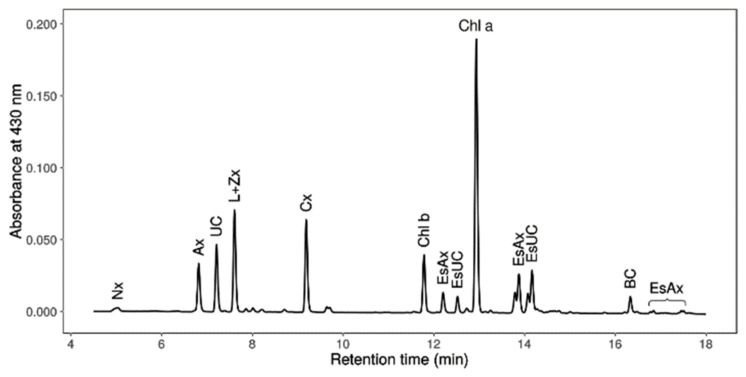
Chromatogram after 4 days of carotenogenesis. Pigments are the followings. Nx: Neoxanthin, Ax: Free astaxanthin, UC: Unknown carotenoid, L+Zx: Lutein + Zeaxanthin, Cx: Canthaxanthin, Chl b: Chlorophyll b, EsAx: Esterified astaxanthin, EsUC: Esterified Unknown carotenoid, Chl a: Chlorophyll a, and BC: β-carotene.

Moreover, *Coelastrella* sp. S6 accumulated free and esterified astaxanthin as well as canthaxanthin (Figure 7). Its carotenogenesis biosynthetic pathway thus seems different from that of *H. pluvialis,* which produces mostly esterified astaxanthin [59], and seems close to the carotenogenesis of *C. zofingiensis*, *Chlorococcum* sp., and other *Coelastrella* strains, which accumulate the same carotenoids species as *Coelastrella* sp. S6 [16,17,60,61,62]. The biosynthesis pathway of astaxanthin and canthaxanthin has only been recently elucidated in *C. zofingiensis* and showed that astaxanthin is synthesized from the ketolation of zeaxanthin, rather than by the hydroxylation of canthaxanthin [63], setting the basis for rational genetic engineering. Such a pathway would be also worth investigating in *Coelastrella* sp. S6 in order to determine if it is conserved between the two species.

#### 3.2.7. Fatty Acid and Lipid Contents of *Coelastrella* sp. S6 in the Light

Astaxanthin production is usually accompanied by fatty acid accumulation, which is required for astaxanthin esterification and additionally used as electron and carbon sink under photo-oxidative stress [6]. Fatty acid content and composition in *Coelastrella* sp. S6 were investigated at the beginning of the carotenogenesis induction (day 0), after 4 days (day 4) and at the end (day 15) of the “red” phase (see Table 3), using the methodology described in Section 2.8 of the Materials and Methods Section. The major fatty acids accumulated during the carotenogenesis, as determined by FAMEs analysis, were palmitic (C16:0), cis-oleic (C18:1n9c), and α-linolenic (C18:3n3) acids, as in other *Coelastrella* species [16,57,61,64,65].

At day 0, the total fatty acids (TFAs) were 3 times more abundant after a growth phase in the “CO_2_” condition (~15% DW) compared to the “Air” condition, which displayed values (i.e., ~5% DW) consistent with those obtained for other green microalgae [56,66]. The “Air” condition favors the formation of polyunsaturated fatty acids (PUFAs), whose proportion in TFAs accounts for ~46% (vs. ~30% for the “CO_2_” condition). The higher amount of TFAs at day 0 for the CO_2_ condition could be explained, on the one hand, because the “CO_2_” green condition induces a shift in the algal metabolism towards the accumulation and storage of lipids, and on the other hand, because the natural nitrate depletion linked to fast growth can “boost” the fatty acid production to esterify astaxanthin already produced during the stationary phase of the growth phase. The amount of TFAs gradually increased during carotenogenesis and, at day 15, the TFA content stabilized at ~30% DW for both conditions. We also see a decrease of PUFAs (~30% against ~46% TFAs) at day 15 and an increase of MUFAs (~38 against ~25% DW) compared to day 0. An increase of MUFAs under nitrogen starvation (~12% TFAs in -N condition against 33% TFAs) has been recently described in *Coelastrella multistriata* MZ Ch23 [12].

The presence of CO_2_ in the growth phase also has a positive impact on the total lipid content as determined by a gravimetric analysis [44] since ~35% DW of lipid accumulated for the “CO_2_” condition at day 0 (vs. ~25% DW for the “Air” condition) (see Table 3). This difference is probably due to a modification of the algal metabolism towards carbon storage, as already discussed. It vanished after 15 days of carotenogenesis since both conditions led to the accumulation of ~40% of total lipids per DW. In conclusion, these analyses showed that *Coelastrella* sp. S6 may be considered as an oleaginous microalga as it shows an average total lipid content of ~25 and ~43% DW in non-stress and stress conditions, respectively [67].

#### 3.2.8. Fatty Acid and Lipid Contents of *Coelastrella* sp. S6 under Heterotrophic Conditions

Fatty acid profile and contents in heterotrophy are described in Table 4, at the beginning (day 0), at day 4, and at day 15 of the induction of carotenogenesis. Condition 2 induced an increase of palmitic (C16:0) and cis-oleic (C18:1n9c) acids compared to Condition 1, with cis-oleic acids reaching ~62 mg·g^−1^ DW at day 15 (Table 4). These results suggest that Condition 2 favors the accumulation of saturated and monounsaturated fatty acids in the dark.

Heterotrophic green growth on glucose led to the accumulation of 6.2 ± 0.2% DW of total fatty acids (TFAs) at day 0, which is consistent with values obtained in other microalgae [47,56], and this value did not change upon the stress of Condition 1. In contrast, the TFA content was increased more than twice in Condition 2, reaching 13.2% DW at day 15, and the TFA fraction was enriched in MUFAs, which is the most abundant class (~50% TFAs) in this condition. This increase of TFAs is expected, considering the accumulation of astaxanthin and canthaxanthin in Condition 2 (Table 2).

The same conclusion can be made by analyzing the total lipid content, which did not increase much in Condition 1 during stress (~28 against ~22% DW), whereas Condition 2 led to the accumulation of up to ~40% DW lipids at day 15.

## 4. Discussion

This paper describes a strain of *Coelastrella*, named *Coelastrella* sp. S6, isolated in the surroundings of Liège, Belgium. This isolate presents a rapid growth (µmax = 2.25 d^−1^), when CO_2_ is added at an incident irradiance of 400 µmol·m^−2^·s^−1^. To our knowledge, this represents a rapid division rate, not yet reported for any other *Coelastrella* strains (µ = 0.3 d^−1^ in [61], µ = 0.95 d^−1^ in [13], µ = 0.25 to 0.37 d^−1^ in [20], µ = 0.56 d^−1^ in [8], and 0.15 d^−1^ in [68]). The growth rate of *Coelastrella* sp. S6 is also interesting compared to that of *H. pluvialis* strains, which usually divide slowly (e.g., µ = 0.41 d^−1^ in [69], µ = 0.57 d^−1^ in [70]) and even compared to the *H. pluvialis* strain with rapid growth under optimized conditions (µ = 1.30 d^−1^ [71]).

*Coelastrella* sp. S6 synthesizes two main, secondary pigments, astaxanthin and canthaxanthin, as do other *Coeslastrella* strains and *C. zofingiensis* [6,13,68]. Table 5 presents the contents of astaxanthin and canthaxanthin in a few *Coelastrella* and *C. zofingiensis* species, taken as examples for the accumulation of these pigments. The values obtained in phototrophy (1.7–1.8 mg·g^−1^ DW for astaxanthin and 1.5–2 mg mg·g^−1^ DW for canthaxanthin) at the end of the red phase are not the highest recorded, but they are in the range of those found in some of the strains cited, even though the conditions are different, and comparisons are thus difficult. However, they do not compete with those found in *Haematococcus* (Table 5). The cumulated contents of astaxanthin and canthaxanthin of *Coelastrella* sp. S6 are similar for both the “Air” and the “CO_2_” growth conditions (3.3 mg·g^−1^ DW and 3.7 mg·g^−1^ DW, respectively). However, it is worth noting that the corresponding productivities are 4 and 5 times higher, respectively, for cells cultivated under the growth “Air” condition than for the cells cultivated under the “CO_2_” condition because the cells are still dividing, even under nitrogen depletion.

The results presented here also show that *Coelastrella* sp. S6 is able to accumulate astaxanthin in the dark. The content is extremely low when nitrogen starvation is applied alone and increases by 2 times when nitrogen starvation is combined with ROS induction by the addition of menadione sodium bisulfite, acetate, and Fe^2+^. The addition of acetate in combination with Fe^++^ has been shown to promote carotenogenesis in the light in *H. pluvialis* [9]. Menadione sodium bisulfite, whose main target would be mitochondria in mammal cells [74], is a naphthoquinone that becomes an unstable semiquinone radical that can enter in a redox cycle inside the cell [75,76]. Another combination of stress, e.g., nitrogen starvation and H_2_O_2_ 0.1 mM with Fe^++^ 18 μM, was also tried, but this did not lead to increased amounts of astaxanthin and canthaxanthin compared to nitrogen starvation alone (0.10 ± 0.02 against 0.14 ± 0.02 mg·g^−1^ DW for astaxanthin and 0.034 ± 0.004 against 0.029 ± 0.005 mg·g^−1^ DW for canthaxanthin, contents at day 15 for nitrogen starvation alone and addition of H_2_O_2_, respectively). Astaxanthin production in heterotrophy has been reported for *C. zofingiensis*, where the content has been optimized by increasing the C/N ratio, and reaches 0.95 mg·g^−1^ DW [77]. Although the content of *Coelastrella* sp. S6 in heterotrophy is much lower (0.20 and 0.13 for astaxanthin and canthaxanthin respectively), it is close to that of the fungus *Xanthophyllomyces dendrorhous* (0.2–0.4 mg·g^−1^ DW), which naturally produces astaxanthin [1]. Astaxanthin production of *Coelastrella* sp. S6 could be increased by optimizing cultivation conditions [78] and/or metabolic engineering as it was done in *X. dendrorhous* [1]. The link between oxidative stress in the dark and astaxanthin accumulation would be worth investigating. As a matter of fact, the alternative pathway of respiration, which is responsible for lowering ROS production of the mitochondrial electron chain [79], has been proposed to control the expression of the β-carotenoid ketolase (BKT) and β-carotenoid hydroxylase (CHYb) genes in *C. zofingiensis* [80].

The results presented here also show that *Coelastrella* sp. S6 is a good producer of TFAs, with ~30% DW at the end of the reddening phase in phototrophy, comprising a majority of SFAs and MUFAs (~68% TFAs), which are considered as good precursors for biodiesel [81]. Reddening in heterotrophy also causes a significant increase of TFAs, at least in Condition 2, which account for ~13% DW. Considering the fatty acid profile and content in this condition (Table 4), a clear increase of palmitic acid (C16:0) and oleic acid (C18:1) is noted when comparing Condition 1 and 2 at day 15 of the reddening phase. This increase could be related to the inhibition of desaturases. Indeed, a decrease of the transcript levels of desaturases has been reported after menadione sodium bisulfite treatment of the fungus *Aspergillus oryzae* [82]. Since desaturases are oxygen-dependent enzymes [83], this increase could also be due to a limited intra-cellular oxygen level in heterotrophy. However, Condition 1 does not lead to such an increase, which suggests that the fatty acid profile modification of Condition 2 is indeed due to the specific action of menadione sodium bisulfite. Furthermore, at the end of the phototrophic growth phase under “Air”, although the amount of TFAs is low (~5% DW), cells accumulated PUFAs in large proportions (~45% TFAs), which are interesting for human health [67]. Finally, the total lipid content of *Coelastrella* sp. S6 is comparable to that of a recently characterized *Coelastrella* species [12]) (Table 6).

In conclusion, the very rapid phototrophic growth conditions determined for *Coelastrella* sp. S6 makes this isolate an interesting candidate for biorefinery purposes where both low-value (lipids) and high-value (astaxanthin and canthaxanthin) products could be valorized, improving the economic viability of this alga. Future prospects should be oriented towards the development of cultivation strategies, allowing the maximization of both compounds. Furthermore, the results presented here show, for the first time to our knowledge, the possibility of heterotrophic astaxanthin and canthaxanthin accumulation for a *Coelastrella* strain, a path that should also be subjected to further optimization.

## Figures and Tables

**Table 1:** Summary of the growth conditions used.

Phase 1: Growth					Phase 2: Carotenogenesis			
Phototrophy	High Light	Air	Condition “Air”	7 days	High Light + 5% CO_2_ + Nitrate starvation			15 days
		5% CO_2_	Condition “CO_2_”					
Heterotrophy	Dark + Glucose + Air		/	10 days	Dark + Glucose + Air + Nitrate starvation	/	Condition 1	
						+ Acetate + MSB + Fe^2+^	Condition 2	

**Table 2:** Astaxanthin and canthaxanthin content (mg·g^−1^ DW), and yield (mg·L^−1^) after 15 days of carotenogenesis in different conditions. The data are reported as the mean ± standard deviation from three independent biological replicates.

		Content (mg·g^−1^ DW)	Yield (mg·L^−1^)
Astaxanthin			
**Phototrophy**	**Air**	1.68 ± 0.27	12.19 ± 1.78
	**CO_2_**	1.83 ± 0.11	3.99 ± 0.35
**Heterotrophy**	**Condition 1**	0.120 ± 0.020	0.102 ± 0.040
	**Condition 2**	0.200 ± 0.030	0.180 ± 0.030
Canthaxanthin			
**Phototrophy**	**Air**	2.04 ± 0.12	14.81 ± 1.11
	**CO_2_**	1.46 ± 0.07	3.18 ± 0.31
**Heterotrophy**	**Condition 1**	0.031 ± 0.003	0.030 ± 0.001
	**Condition 2**	0.130 ± 0.010	0.114 ± 0.010

**Table 3:** Fatty acid profile and quantification in phototrophic conditions. Day 0: end of the growth phase; day 4: after 4 days of stress; day 15: after 15 days of stress. SFAs = saturated fatty acids, MUFAs = monounsaturated fatty acids, PUFAs = polyunsaturated fatty acids, and TFAs = total fatty acids. The fatty acids are expressed in mg·g^−1^ DW. The SFA, MUFA, and PUFA classes are expressed in %TFAs. The TFAs and total lipids are expressed in %DW. The data are reported as the mean ± standard deviation from three independent biological replicates.

	Day 0		Day 4		Day 15	
	Air	CO_2_	Air	CO_2_	Air	CO_2_
C16:0	8.3 ± 1.1	37.8 ± 4	81.2 ± 5.6	70.8 ± 1.9	94.4 ± 3.6	103 ± 6.1
C18:0	1.7 ± 0.2	6.6 ± 0.5	7.3 ± 0.5	6.4 ± 0.8	7.2 ± 0.3	7.8 ± 0.4
C18:1n9c	7.9 ± 1.1	46.3 ± 5.1	121.9 ± 8.3	72.6 ± 3.8	120.8 ± 4.4	91.9 ± 4.7
C18:2n6c	4.3 ± 0.5	13.8 ± 1.8	23.4 ± 1.4	17.9 ± 0.2	27.5 ± 1	23.8 ± 1.2
C18:3n3	16.9 ± 1.8	29.4 ± 4.4	53.1 ± 3.8	54.7 ± 0.4	73.2 ± 3	75.6 ± 3.6
C20:0	1.1 ± 0.1	1.9 ± 0.2	2.9 ± 0.1	2.8 ± 0.1	3.3 ± 0.1	3.5 ± 0.1
SFAs	29.9 ± 1.9	36.1 ± 0.3	31.8 ± 0.1	36.4 ± 0.4	32.5 ± 0.1	38.2 ± 0.3
MUFAs	24.5 ± 0.5	34.4 ± 1.4	42.7 ± 0.1	32.7 ± 0.8	37.6 ± 0.2	30.5 ± 0.8
PUFAs	45.6 ± 1.9	29.5 ± 1.6	25.6 ± 0	30.9 ± 0.8	30 ± 0.2	31.4 ± 0.6
TFAs	4.6 ± 0.5	14.9 ± 1.6	29.9 ± 2	23.8 ± 0.6	33.6 ± 1.3	32 ± 1.6
Total Lipids	24.8 ± 3	35.3 ± 2.2	-	-	44.2 ± 0.7	42.6 ± 0.7

**Table 4:** Fatty acid profile and quantification in heterotrophic conditions. Day 0: end of the growth phase; day 4: after 4 days of stress; day 15: after 15 days of stress. SFAs = saturated fatty acids, MUFAs = monounsaturated fatty acids, PUFAs = polyunsaturated fatty acids, and TFAs = total Fatty acids. The fatty acids are expressed in mg·g^−1^ DW. The SFA, MUFA, and PUFA classes are expressed in %TFAs. The TFAs and total lipids are expressed in %DW. The data are presented as the mean ± standard deviation from three independent biological replicates.

	Day 0	Day 4		Day 15	
	Heterotrophy	Condition 1	Condition 2	Condition 1	Condition 2
C16:0	18.8 ± 0.7	18.5 ± 1	26.2 ± 1.5	20.3 ± 0.3	35.2 ± 1.6
C16:1	-	1.6 ± 0.3	2.3 ± 0.2	1.6 ± 0.3	3.7 ± 0.4
C18:0	10.2 ± 0.4	6.2 ± 0.3	8.4 ± 0.6	5.7 ± 0.1	10.4 ± 0.8
C18:1n9c	16.4 ± 0.7	22 ± 0.9	39.3 ± 2.8	23.8 ± 0.3	62.2 ± 6.4
C18:2n6c	6.9 ± 0.1	6.9 ± 0.9	7.7 ± 0.5	6.5 ± 0	10.8 ± 0.9
C18:3n3	4.1 ± 0.3	4.9 ± 0.5	6.4 ± 0.3	4.4 ± 0.4	6.2 ± 0.3
C20:0	2.2 ± 0.3	1.2 ± 0.2	1.4 ± 0.2	1.1 ± 0.1	1.6 ± 0.1
SFAs	51.9 ± 0.9	42.2 ± 0.5	38.1 ± 2.7	43.8 ± 0.3	36.9 ± 0.9
MUFAs	28.6 ± 0.6	38.6 ± 0.5	44.2 ± 4.2	39.5 ± 0.3	50.3 ± 1.1
PUFAs	19.5 ± 0.3	19.2 ± 0.9	14.9 ± 1.1	16.8 ± 0.6	12.9 ± 0.2
TFAs	6.2 ± 0.2	6.2 ± 0.4	9.2 ± 0.6	6.5 ± 0	13.2 ± 1.1
Total Lipids	22 ± 0.6	-	-	27.8 ± 0	39.5 ± 0.9

**Table 5:** Astaxanthin and canthaxanthin content (mg·g^−1^ DW) in some species of *Coelastrella*, *C. zofingiensis* and *H. pluvialis* strains.

Strain	Growth Phase	Stress Condition					
		Nutrient Stress and Addition	Light Intensity (µmol·m^−2^·s^−1^)	Duration (Days)	Astaxanthin	Canthaxanthin	References
*Coelastrella* sp. S6	Phototrophy—5% CO_2_	N starvation + 5% CO_2_	400	15	1.83 ± 0.11	1.46 ± 0.07	This manuscript
	Phototrophy (Air)	N starvation + 5% CO_2_	400	15	1.68 ± 0.27	2.04 ± 0.12	
	Heterotrophy	N starvation + MSB + Fe^2+^ + Acetate	0	15	0.20 ± 0.03	0.13 ± 0.01	
*Coelastrella striolata* var. *multistriata*	Phototrophy	Aging	40	21	1	35	[62]
		Aging	65	50	1.5	47.5	[61]
		Aging	214	21	0.99 ± 0.21	0.42 ± 0.05	[68]
*Coelastrella* sp. F50	Phototrophy	1.5% NaCl	400	12	18	1.8	[47]
*Coelastrella rubescens*	Phototrophy	N and P starvation, low pH	240	8	7.95 ± 0.85	12.73 ± 0.56	[13] ^a^
*C. zofingiensis*	Phototrophy—High Light	High C:N	> 80	12	1.80 ± 0.10	-	[72]
	Phototrophy—Low light	High C:N	> 80	12	2.19 ± 0.19	-	
*H. pluvialis*	Phototrophy	N starvation	350	12	21.8 ± 0.4	-	[73]

^a^ highest values.

**Table 6:** Lipid content of *Coelastrella* sp S6 and *C. striolata* MZ-Ch23.

Strain	Stress Condition	Light Intensity (µmol·m^−2^·s^−1^)	CO_2_ Addition (%) or Glucose (g·L^−1^)	Duration	Lipids (% DW)	References
Green cells						
*Coelastrella* sp. S6	-	400 (Air)	-	7	24.8 ± 0.3	This manuscript
	-	400 (CO_2_)	5%	7	35.3 ± 2.2	
	-	0	10 g·L^−1^	10	22.0 ± 0.6	
*Coelastrella striolata MZ-Ch23*	-	100 (16:8 L:D)	-	25	27.0 ± 3.64	[12]
Red cells						
*Coelastrella* sp. S6	N-starvation	400 (Air)	5%	15	44.2 ± 0.7	This manuscript
	N-starvation	400 (CO_2_)	5%	15	42.6 ± 0.7	
	N-starvation	-	10 g·L^−1^	15	27.8 ± 0	
	N-starvation + MSB + acetate + Fe^2+^	-	10 g·L^−1^	15	39.5 ± 0.9	
*Coelastrella striolata MZ-Ch23*	N-starvation	100 (16:8 L:D)	-	25	45.90 ± 1.67	[12]

## Data Availability

Not applicable.

## References

[1] Torres-Haro A., Verdín J., Kirchmayr M.R., Arellano-Plaza M. (2021). Metabolic Engineering for High Yield Synthesis of Astaxanthin in *Xanthophyllomyces dendrorhous*. Microb. Cell Fact..

[2] Mota G.C.P., de Moraes L.B.S., Oliveira C.Y.B., Oliveira D.W.S., de Abreu J.L., Dantas D.M.M., Gálvez A.O. (2021). Astaxanthin from *Haematococcus pluvialis*: Processes, Applications, and Market. Prep. Biochem. Biotechnol..

[3] Lorenz R.T., Cysewski G.R. (2000). Commercial Potential for *Haematococcus* Microalgae as a Natural Source of Astaxanthin. Trends Biotechnol..

[4] Wang H.-Q., Sun X.-B., Xu Y.-X., Zhao H., Zhu Q.-Y., Zhu C.-Q. (2010). Astaxanthin Upregulates Heme Oxygenase-1 Expression through ERK1/2 Pathway and Its Protective Effect against Beta-Amyloid-Induced Cytotoxicity in SH-SY5Y Cells. Brain Res..

[5] Rebelo B.A., Farrona S., Ventura M.R., Abranches R. (2020). Canthaxanthin, a Red-Hot Carotenoid: Applications, Synthesis, and Biosynthetic Evolution. Plants.

[6] Lemoine Y., Schoefs B. (2010). Secondary Ketocarotenoid Astaxanthin Biosynthesis in Algae: A Multifunctional Response to Stress. Photosynth. Res..

[7] Mariam I., Kareya M.S., Rehmanji M., Nesamma A.A., Jutur P.P. (2021). Channeling of Carbon Flux Towards Carotenogenesis in *Botryococcus braunii*: A Media Engineering Perspective. Front. Microbiol..

[8] Minhas A.K., Hodgson P., Barrow C.J., Adholeya A. (2020). Two-Phase Method of Cultivating Coelastrella Species for Increased Production of Lipids and Carotenoids. Bioresour. Technol. Rep..

[9] Kobayashi M., Kakizono T., Nagai S. (1993). Enhanced Carotenoid Biosynthesis by Oxidative Stress in Acetate-Induced Cyst Cells of a Green Unicellular Alga, *Haematococcus pluvialis*. Appl. Environ. Microbiol..

[10] Fučíková K., Lewis L.A. (2012). Intersection of *Chlorella*, *Muriella* and *Bracteacoccus*: Resurrecting the Genus *Chromochloris* Kol et Chodat (Chlorophyceae, Chlorophyta). Fottea.

[11] Liu J., Sun Z., Gerken H., Liu Z., Jiang Y., Chen F. (2014). *Chlorella zofingiensis* as an Alternative Microalgal Producer of Astaxanthin: Biology and Industrial Potential. Mar. Drugs.

[12] Maltsev Y., Krivova Z., Maltseva S., Maltseva K., Gorshkova E., Kulikovskiy M. (2021). Lipid Accumulation by *Coelastrella multistriata* (Scenedesmaceae, Sphaeropleales) during Nitrogen and Phosphorus Starvation. Sci. Rep..

[13] Minyuk G., Chelebieva E., Chubchikova I., Dantsyuk N., Drobetskaya I., Sakhon E., Chekanov K., Solovchenko A. (2017). Stress-Induced Secondary Carotenogenesis in *Coelastrella rubescens* (Scenedesmaceae, Chlorophyta), a Producer of Value-Added Keto-Carotenoids. Algae.

[14] Ip P.-F., Chen F. (2005). Production of Astaxanthin by the Green Microalga *Chlorella zofingiensis* in the Dark. Process Biochem..

[15] Hu J., Nagarajan D., Zhang Q., Chang J.S., Lee D.J. (2018). Heterotrophic Cultivation of Microalgae for Pigment Production: A Review. Biotechnol. Adv..

[16] Goecke F., Noda J., Paliocha M., Gislerød H.R. (2020). Revision of *Coelastrella* (Scenedesmaceae, Chlorophyta) and First Register of This Green Coccoid Microalga for Continental Norway. World J. Microbiol. Biotechnol..

[17] Wang Q., Song H., Liu X., Zhu H., Hu Z., Liu G. (2019). Deep Genomic Analysis of *Coelastrella saipanensis* (Scenedesmaceae, Chlorophyta): Comparative Chloroplast Genomics of Scenedesmaceae. Eur. J. Phycol..

[18] Wang Q., Song H., Liu X., Liu B., Hu Z., Liu G. (2019). Morphology and Molecular Phylogeny of Coccoid Green Algae *Coelastrella* Sensu Lato (Scenedesmaceae, Sphaeropeales), Including the Description of Three New Species and Two New Varieties. J. Phycol..

[19] Kawasaki S., Yamazaki K., Nishikata T., Ishige T., Toyoshima H., Miyata A. (2020). Photooxidative Stress-Inducible Orange and Pink Water-Soluble Astaxanthin-Binding Proteins in Eukaryotic Microalga. Commun. Biol..

[20] Minhas A.K., Hodgson P., Barrow C.J., Sashidhar B., Adholeya A. (2016). The Isolation and Identification of New Microalgal Strains Producing Oil and Carotenoid Simultaneously with Biofuel Potential. Bioresour. Technol..

[21] Zaytseva A., Chekanov K., Zaytsev P., Bakhareva D., Gorelova O., Kochkin D., Lobakova E. (2021). Sunscreen Effect Exerted by Secondary Carotenoids and Mycosporine-like Amino Acids in the Aeroterrestrial Chlorophyte *Coelastrella rubescens* under High Light and UV-A Irradiation. Plants.

[22] Minhas A.K., Hodgson P., Barrow C.J., Adholeya A. (2016). A Review on the Assessment of Stress Conditions for Simultaneous Production of Microalgal Lipids and Carotenoids. Front. Microbiol..

[23] Blasio M., Balzano S. (2021). Fatty Acids Derivatives from Eukaryotic Microalgae, Pathways and Potential Applications. Front. Microbiol..

[24] Ziganshina E.E., Bulynina S.S., Ziganshin A.M. (2020). Comparison of the Photoautotrophic Growth Regimens of *Chlorella sorokiniana* in a Photobioreactor for Enhanced Biomass Productivity. Biology.

[25] Bold H.C. (1942). The Cultivation of Algae. Bot. Rev..

[26] Minyuk G.S., Dantsyuk N.V., Chelebieva E.S., Chubchikova I.N., Drobetskaya I.V., Solovchenko A.E. (2019). The Effect of Diverse Nitrogen Sources in the Nutrient Medium on the Growth of the Green Microalgae *Chromochloris zofingiensis* in the Batch Culture. Mar. Biol. J..

[27] Suzuki H., Hulatt C.J., Wijffels R.H., Kiron V. (2019). Growth and LC-PUFA Production of the Cold-Adapted Microalga *Koliella antarctica* in Photobioreactors. J. Appl. Phycol..

[28] Newman S.M., Boynton J.E., Gillham N.W., Randolph-Anderson B.L., Johnson A.M., Harris E.H. (1990). Transformation of Chloroplast Ribosomal RNA Genes in Chlamydomonas: Molecular and Genetic Characterization of Integration Events. Genetics.

[29] Ferrigo D., Galla G., Sforza E., Morosinotto T., Barcaccia G., Ceschi Berrini C. (2015). Biochemical Characterization and Genetic Identity of an Oil-Rich *Acutodesmus obliquus* Isolate. J. Appl. Phycol..

[30] Huang X., Madan A. (1999). CAP3: A DNA Sequence Assembly Program. Genome Res..

[31] Altschul S.F., Gish W., Miller W., Myers E.W., Lipman D.J. (1990). Basic Local Alignment Search Tool. J. Mol. Biol..

[32] Benson D.A. (2000). GenBank. Nucleic Acids Res..

[33] Katoh K., Standley D.M. (2013). MAFFT Multiple Sequence Alignment Software Version 7: Improvements in Performance and Usability. Mol. Biol. Evol..

[34] Katoh K., Standley D.M. (2016). A Simple Method to Control Over-Alignment in the MAFFT Multiple Sequence Alignment Program. Bioinformatics.

[35] Gouy M., Guindon S., Gascuel O. (2010). SeaView Version 4: A Multiplatform Graphical User Interface for Sequence Alignment and Phylogenetic Tree Building. Mol. Biol. Evol..

[36] Gascuel O. (1997). BIONJ: An Improved Version of the NJ Algorithm Based on a Simple Model of Sequence Data. Mol. Biol. Evol..

[37] Tavaré S. (1986). Some Probabilistic and Statistical Problems in the Analysis of DNA Sequences. Some Mathematical Questions in Biology: DNA Sequence Analysis.

[38] Anisimova M., Gascuel O. (2006). Approximate Likelihood-Ratio Test for Branches: A Fast, Accurate, and Powerful Alternative. Syst. Biol..

[39] Letunic I., Bork P. (2019). Interactive Tree of Life (ITOL) v4: Recent Updates and New Developments. Nucleic Acids Res..

[40] Chi N.T.L., Duc P.A., Mathimani T., Pugazhendhi A. (2019). Evaluating the Potential of Green Alga Chlorella Sp. for High Biomass and Lipid Production in Biodiesel Viewpoint. Biocatal. Agric. Biotechnol..

[41] Cataldo D.A., Maroon M., Schrader L.E., Youngs V.L. (1975). Rapid Colorimetric Determination of Nitrate in Plant Tissue by Nitration of Salicylic Acid. Commun. Soil Sci. Plant Anal..

[42] Beckers L., Hiligsmann S., Hamilton C., Masset J., Thonart P. (2010). Fermentative Hydrogen Production by *Clostridium butyricum* CWBI1009 and Citrobacter Freundii CWBI952 in Pure and Mixed Cultures. Biotechnol. Agron. Soc. Environ..

[43] Gérin S., Leprince P., Sluse F.E., Franck F., Mathy G. (2016). New Features on the Environmental Regulation of Metabolism Revealed by Modeling the Cellular Proteomic Adaptations Induced by Light, Carbon, and Inorganic Nitrogen in Chlamydomonas Reinhardtii. Front. Plant Sci..

[44] Bligh E.G., Dyer W.J. (1959). A Rapid Method of Total Lipid Extraction and Purification. Can. J. Biochem. Physiol..

[45] Gérin S., Delhez T., Corato A., Remacle C., Franck F. (2020). A Novel Culture Medium for Freshwater Diatoms Promotes Efficient Photoautotrophic Batch Production of Biomass, Fucoxanthin, and Eicosapentaenoic Acid. J. Appl. Phycol..

[46] Neofotis P., Huang A., Sury K., Chang W., Joseph F., Gabr A., Twary S., Qiu W., Holguin O., Polle J.E.W. (2016). Characterization and Classification of Highly Productive Microalgae Strains Discovered for Biofuel and Bioproduct Generation. Algal Res..

[47] Hu C.-W., Chuang L.-T., Yu P.-C., Chen C.-N.N. (2013). Pigment Production by a New Thermotolerant Microalga Coelastrella Sp. F50. Food Chem..

[48] Kaufnerová V., Eliáš M. (2013). The Demise of the Genus *Scotiellopsis* Vinatzer (Chlorophyta). Nova Hedwig..

[49] Xie Y., Ho S.-H., Chen C.-N.N., Chen C.-Y., Ng I.-S., Jing K.-J., Chang J.-S., Lu Y. (2013). Phototrophic Cultivation of a Thermo-Tolerant *Desmodesmus* sp. for Lutein Production: Effects of Nitrate Concentration, Light Intensity and Fed-Batch Operation. Bioresour. Technol..

[50] Ho S.-H., Chen C.-Y., Chang J.-S. (2012). Effect of Light Intensity and Nitrogen Starvation on CO_2_ Fixation and Lipid/Carbohydrate Production of an Indigenous Microalga *Scenedesmus obliquus* CNW-N. Bioresour. Technol..

[51] Chekanov K., Schastnaya E., Solovchenko A., Lobakova E. (2017). Effects of CO_2_ Enrichment on Primary Photochemistry, Growth and Astaxanthin Accumulation in the Chlorophyte *Haematococcus pluvialis*. J. Photochem. Photobiol. B Biol..

[52] Kang C.D., Lee J.S., Park T.H., Sim S.J. (2005). Comparison of Heterotrophic and Photoautotrophic Induction on Astaxanthin Production by *Haematococcus pluvialis*. Appl. Microbiol. Biotechnol..

[53] Kaewkannetra P., Enmak P., Chiu T. (2012). The Effect of CO_2_ and Salinity on the Cultivation of *Scenedesmus obliquus* for Biodiesel Production. Biotechnol. Bioprocess Eng..

[54] Singh S.P., Singh P. (2014). Effect of CO_2_ Concentration on Algal Growth: A Review. Renew. Sustain. Energy Rev..

[55] De Marchin T., Erpicum M., Franck F. (2015). Photosynthesis of Scenedesmus Obliquus in Outdoor Open Thin-Layer Cascade System in High and Low CO_2_ in Belgium. J. Biotechnol..

[56] Thanh T.L. (2019). Effects of Carbon Trophic Mode on Growth, Pigments and Fatty Acids in Green Microalgae Analyzed on the Basis of Well-Defined Growth Kinetics of Batch Cultures. Ph.D. Thesis.

[57] Aburai N., Sumida D., Abe K. (2015). Effect of Light Level and Salinity on the Composition and Accumulation of Free and Ester-Type Carotenoids in the Aerial Microalga *Scenedesmus* sp. (Chlorophyceae). Algal Res..

[58] Chekanov K., Lukyanov A., Boussiba S., Aflalo C., Solovchenko A. (2016). Modulation of Photosynthetic Activity and Photoprotection in *Haematococcus pluvialis* Cells during Their Conversion into Haematocysts and Back. Photosynth. Res..

[59] Han D., Li Y., Hu Q. (2013). Astaxanthin in Microalgae: Pathways, Functions and Biotechnological Implications. Algae.

[60] Liu B.-H., Lee Y.-K. (2000). Secondary Carotenoids Formation by the Green Alga *Chlorococcum* sp.. J. Appl. Phycol..

[61] Abe K., Hattori H., Hirano M. (2007). Accumulation and Antioxidant Activity of Secondary Carotenoids in the Aerial Microalga *Coelastrella striolata* Var. *multistriata*. Food Chem..

[62] Abe K., Takizawa H., Kimura S., Hirano M. (2004). Characteristics of Chlorophyll Formation of the Aerial Microalga *Coelastrella striolata* Var. *multistriata* and Its Application for Environmental Biomonitoring. J. Biosci. Bioeng..

[63] Zhang Y., Ye Y., Ding W., Mao X., Li Y., Gerken H., Liu J. (2020). Astaxanthin Is Ketolated from Zeaxanthin Independent of Fatty Acid Synthesis in *Chromochloris zofingiensis*. Plant Physiol..

[64] Minyuk G., Sidorov R., Solovchenko A. (2020). Effect of Nitrogen Source on the Growth, Lipid, and Valuable Carotenoid Production in the Green Microalga *Chromochloris zofingiensis*. J. Appl. Phycol..

[65] Minyuk G.S., Chelebieva E.S., Chubchikova I.N., Dantsyuk N.V., Drobetskaya I.V., Sakhon E.G., Chivkunova O.B., Chekanov K.A., Lobakova E.S., Sidorov R.A. (2016). PH and CO_2_ Effects on *Coelastrella* (Scotiellopsis) Rubescens Growth and Metabolism. Russ. J. Plant Physiol..

[66] Sun X.-M., Ren L.-J., Zhao Q.-Y., Ji X.-J., Huang H. (2018). Microalgae for the Production of Lipid and Carotenoids: A Review with Focus on Stress Regulation and Adaptation. Biotechnol. Biofuels.

[67] Hu Q., Sommerfeld M., Jarvis E., Ghirardi M., Posewitz M., Seibert M., Darzins A. (2008). Microalgal Triacylglycerols as Feedstocks for Biofuel Production: Perspectives and Advances. Plant J..

[68] Aburai N., Ohkubo S., Miyashita H., Abe K. (2013). Composition of Carotenoids and Identification of Aerial Microalgae Isolated from the Surface of Rocks in Mountainous Districts of Japan. Algal Res..

[69] Tocquin P., Fratamico A., Franck F. (2012). Screening for a Low-Cost *Haematococcus pluvialis* Medium Reveals an Unexpected Impact of a Low N/P Ratio on Vegetative Growth. J. Appl. Phycol..

[70] Gong X., Chen F. (1997). Optimization of Culture Medium for Growth of *Haematococcus pluvialis*. J. Appl. Phycol..

[71] Boussiba S. (2000). Carotenogenesis in the Green Alga *Haematococcus pluvialis*: Cellular Physiology and Stress Response. Physiol. Plant.

[72] Chen J., Liu L., Wei D. (2017). Enhanced Production of Astaxanthin by *Chromochloris zofingiensis* in a Microplate-Based Culture System under High Light Irradiation. Bioresour. Technol..

[73] Orosa M., Valero J.F., Herrero C., Abalde J. (2001). Comparison of the Accumulation of Astaxanthin in *Haematococcus pluvialis* and Other Green Microalgae under N-Starvation and High Light Conditions. Biotechnol. Lett..

[74] Loor G., Kondapalli J., Schriewer J.M., Chandel N.S., Vanden Hoek T.L., Schumacker P.T. (2010). Menadione Triggers Cell Death through ROS-Dependent Mechanisms Involving PARP Activation without Requiring Apoptosis. Free Radic. Biol. Med..

[75] Castro F.A.V., Mariani D., Panek A.D., Eleutherio E.C.A., Pereira M.D. (2008). Cytotoxicity Mechanism of Two Naphthoquinones (Menadione and Plumbagin) in *Saccharomyces cerevisiae*. PLoS ONE.

[76] Criddle D.N., Gillies S., Baumgartner-Wilson H.K., Jaffar M., Chinje E.C., Passmore S., Chvanov M., Barrow S., Gerasimenko O.V., Tepikin A.V. (2006). Menadione-Induced Reactive Oxygen Species Generation via Redox Cycling Promotes Apoptosis of Murine Pancreatic Acinar Cells. J. Biol. Chem..

[77] Chen F. (2006). Methods for Production of Astaxanthin from the Green Microalgae Chlorella in Dark-Heterotrophic Cultures. U.S. Patent.

[78] Paliwal C., Jutur P.P. (2021). Dynamic Allocation of Carbon Flux Triggered by Task-Specific Chemicals Is an Effective Non-Gene Disruptive Strategy for Sustainable and Cost-Effective Algal Biorefineries. Chem. Eng. J..

[79] Umbach A.L., Fiorani F., Siedow J.N. (2005). Characterization of Transformed Arabidopsis with Altered Alternative Oxidase Levels and Analysis of Effects on Reactive Oxygen Species in Tissue. Plant Physiol..

[80] Li Y., Huang J., Sandmann G., Chen F. (2008). Glucose Sensing and the Mitochondrial Alternative Pathway Are Involved in the Regulation of Astaxanthin Biosynthesis in the Dark-Grown *Chlorella zofingiensis* (Chlorophyceae). Planta.

[81] Suriya Narayanan G., Kumar G., Seepana S., Elankovan R., Arumugan S., Premalatha M. (2018). Isolation, Identification and Outdoor Cultivation of Thermophilic Freshwater Microalgae Coelastrella Sp. FI69 in Bubble Column Reactor for the Application of Biofuel Production. Biocatal. Agric. Biotechnol..

[82] Shao H., Tu Y., Wang Y., Jiang C., Ma L., Hu Z., Wang J., Zeng B., He B. (2019). Oxidative Stress Response of Aspergillus Oryzae Induced by Hydrogen Peroxide and Menadione Sodium Bisulfite. Microorganisms.

[83] Shanklin J., Cahoon E.B. (1998). Desaturation and Related Modifications of Fatty Acids1. Annu. Rev. Plant Physiol. Plant Mol. Biol..

